# The Toughness-Enhanced Atelocollagen Double-Network Gel for Biomaterials

**DOI:** 10.3390/polym16020283

**Published:** 2024-01-19

**Authors:** Atsushi Tsuyukubo, Riku Kubota, Yuzo Sato, Ichiro Fujimoto

**Affiliations:** Koken Research Institute, Koken Co., Ltd., 1-18-36 Takarada, Tsuruoka 997-0011, Yamagata, Japan

**Keywords:** atelocollagen, double-network gel, toughness, soft tissue

## Abstract

A tough gel composed of atelocollagen, which lacks an immunogenetic site, is a promising material for biomedical application. In this study, we created a composite hydrogel composed of atelocollagen gel cross-linked with glutaraldehyde (GA) and poly-(*N*,*N*-dimethylacrylamide) gel exhibiting biocompatibility based on the double-network (DN) gel principle. The tensile toughness of atelocollagen gel remained constant regardless of the amount of cross-linker (GA) used. In contrast, tensile tests of the DN gel indicated that mechanical properties, such as fracture stress and toughness, were significantly higher than those of the atelocollagen gel. Moreover, fibroblast cells adhered and spread on the gels, the Schiff bases of which were treated via reductive amination for detoxification from GA. These findings demonstrate the potential of the proposed gel materials as artificial alternative materials to soft tissues with sub-MPa fracture stress.

## 1. Introduction

Collagen is the most abundant natural protein comprising soft tissues in all animals. It has been utilized as a biomaterial for diverse applications, such as cell culture scaffolds and drug delivery systems (DDS) [1,2,3]. Atelocollagen, which is prepared by removing N- and C-terminal telopeptide regions, is also a promising biomaterial for its reduced immunogenicity [4,5]. We have previously demonstrated the potential application of atelocollagen in drug development, DDS, and regenerative medicine [6,7,8,9,10,11]. Most recently, we developed toughness-enhanced atelocollagen threads (i.e., anisotropic hydrogels) using slide-ring polymers, which demonstrated their potential as alternatives to tendon or ligament materials [10,11].

Hydrogels are chemically and physically formed three-dimensional polymer networks that contain large quantities of water. Therefore, hydrogels are soft and flexible materials that can transport, transmit, and exchange water-soluble substances, like soft tissues in the human body. Because a collagen constitutes the majority of organs and tissues in the human body, hydrogels based on collagen or atelocollagen are expected to be applied to biomaterials such as DDS carriers, cell culture scaffolds, and biosensors [12,13,14,15]. However, their weak mechanical strength is a major obstacle to their implementation in biological applications of alternative materials to the soft tissue where strict load is applied, such as tendons and ligaments.

To overcome the limited strength and stretchability of conventional hydrogels, several new classes of hydrogels have been developed by designing polymer network structures strategically [16,17,18,19,20,21,22,23,24,25,26,27,28,29,30,31,32,33]. Especially, a double-network (DN) gel developed by J. P. Gong et al. is one of the approaches used to enhance the mechanical properties for biomedical applications. The DN gel is generally known as an interpenetrating polymer network composed of a stiff and brittle cross-linked polymer network (first network) and a soft and ductile network (second network). DN gels offer high mechanical strength and toughness, which is attributed to effective energy dissipation through the prior fracture of the brittle first network [22,23,25]. This approach to toughen gels can be applied to various synthetic and natural polymer gels as long as the polymer networks do not interfere with each other [34,35,36,37,38]. Moreover, DN gels are applicable to medical research fields such as tissue engineering and drug delivery [39,40]. Therefore, we developed toughness-enhanced atelocollagen gels based on the principle of DN gels mentioned above. Additionally, this material is expected to be applied as an artificial alternative material to soft tissue, which can inhibit undesired immunoreaction after transplantation. To date, such tough atelocollagen gels have hardly been reported.

**Figure 1:**
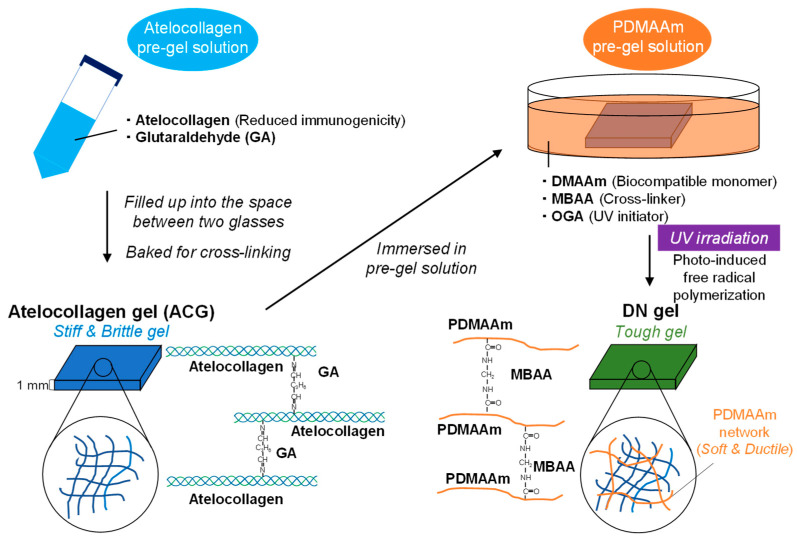
Schematic illustration for preparation of double-network (DN) gel composed of atelocollagen and poly-(*N*,*N*-dimethylacrylamide).

In this study, we developed a composite hydrogel consisting of atelocollagen and a biocompatible polymer based on the DN principle, aiming for artificial alternative materials to soft tissues (Figure 1). Firstly, in order to form a stiff and brittle polymer network, we prepared a chemically cross-linked atelocollagen gel. We selected glutaraldehyde (GA) as a cross-linker for atelocollagen because GA has been mostly utilized in various studies on collagen gels [41,42]. We then combined soft and ductile polymer networks having biocompatibility with this gel. We selected poly-(*N*,*N*-dimethylacrylamide) (PDMAAm), which is a component of a soft contact lens, as the biocompatible polymer. PDMAAm can be cross-linked through free-radical polymerization with a cross-linker containing vinyl groups. First, we investigated the mechanical properties of both the atelocollagen and DN gels using tensile tests. Next, we measured the water content and polymer network structure in the DN gel by evaluating its physical properties by combining two polymer networks. Finally, to evaluate their potential as biomaterials, we observed cell adhesion on the gel and confirmed reduced cytotoxicity.

## 2. Materials and Methods

### 2.1. Materials

A 3 wt% aqueous solution of industrial-grade type I atelocollagen (30 mg/mL) was prepared in our company (KOKEN Co., Ltd., Yamagata, Japan). *N*,*N*-dimethylacrylamide (DMAAm) was purchased from Kanto Chemical Co., Inc. (Tokyo, Japan). Glutaraldehyde (GA), *N*,*N’*-methylenebisacrylamide (MBAA), 2-oxoglutaric acid (OGA), penicillin-streptomycin solution (×100), and sodium cyanoborohydride (NaBH_3_CN) were purchased from Fujifilm Wako Pure Chemical Corporation (Osaka, Japan). Primary Normal Human Dermal Fibroblast (NHDF) and FibroLife comp kit as culture medium for NHDF cells were purchased from KURABO (Osaka, Japan). The V79 cell line, which is a lung fibroblast derived from Chinese hamsters, was purchased from the JCRB cell bank (Osaka, Japan). Calcein-AM was purchased from Dojindo (Kumamoto, Japan). MEM and L-glutamine were purchased from Life Technologies Corporation (Carlsbad, CA, USA). Fetal bovine serum (FBS) was purchased from Cytiva (Tokyo, Japan). Falcon tissue culture plate, 48 wells (flat bottom with low evaporation lid), was purchased from Corning Inc. (Corning, New York, NY, USA).

### 2.2. Preparation of GA Cross-Linked Atelocollagen Gel (GC-ACG) as First Network

Pre-gel aqueous solutions containing 2 wt% atelocollagen and GA as cross-linkers were prepared. The solutions were prepared at various ratios (mol/mol) of GA concentration against those of lysine (Lys) and hydroxylysine (Hyl) residues, which react with the aldehyde group in GA. The solution was degassed under reduced pressure, and the bubbles were removed using a centrifuge machine. It was filled into the 1 mm space between two glasses and baked at 30 °C for 24 h to cross-link atelocollagen with GA. The formed GC-ACGs were immersed in water.

### 2.3. Preparation of DN Gels Composed of Atelocollagen and PDMAAm

GC-ACG, prepared using the method described in Section 2.2 under specific GA concentrations, was soaked in a PDMAAm pre-gel aqueous solution containing 3 M DMAAm as a monomer, MBAA as a cross-linker, and 1 mol% OGA as the UV initiator (the molar percentages are relative to the monomer) at 4 °C for 1 d. This solution was degassed under reduced pressure and purged with N_2_ to remove the dissolved oxygen. The gel was then irradiated with UV light for an hour using an irradiation apparatus (λ = 365 nm, UV cross-linker CL-1000, UVP, Upland, CA, USA) to initiate free radical polymerization of DMAAm and MBAA. After irradiation, the samples were immersed in water. DN gels were prepared with various MBAA concentrations (mol%).

### 2.4. Tensile Tests

**Figure 2:**
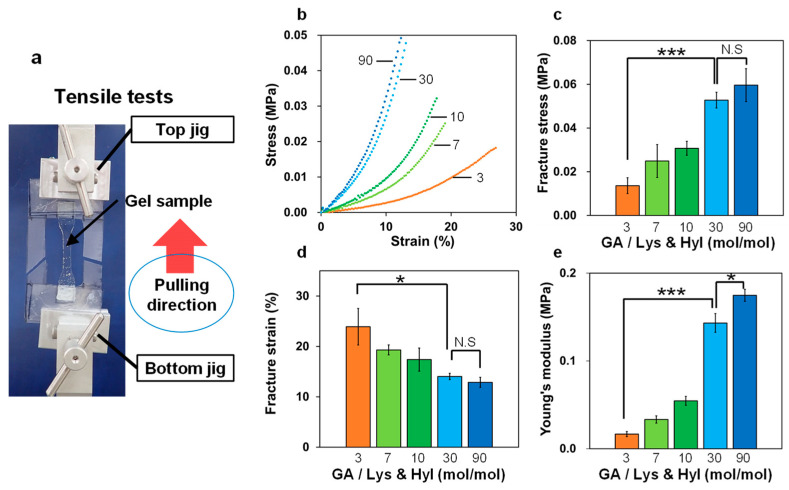
Mechanical tensile properties of glutaraldehyde (GA) cross-linked atelocollagen gels (GC-ACGs) under various ratios of GA/(Lys & Hyl). (**a**) Manner of tensile tests; (**b**) stress–strain curve; (**c**) fracture stress; (**d**) fracture strain; (**e**) Young’s modulus (n = 3; N.S: not significant; * *p* < 0.05; *** *p* < 0.001).

Both the GC-ACG and DN gels were cut out in a dumbbell shape standardized according to the JIS-K6251-8 size. Tensile tests were performed using a mechanical testing instrument (EZ-S, Shimazu Co., Ltd., Kyoto, Japan) with a tensile velocity of 100 mm/min in air at room temperature. All gel samples were glued to two polyethylene terephthalate (PET) films, as illustrated in Figure 2a, and they were measured after immersion in water for 1 d. The tensile fracture stress was determined to be the nominal stress at the breaking point. The toughness, which is the energy required for fracture, was determined from the area under the stress–strain curve.

### 2.5. Measurement of Water Content of Gels

The surface water of the hydrogels was wiped off, and the weight (*W_s_*) of the swollen gels was measured. Next, the swollen gels were freeze-dried at −80 °C for 1 d, and the weight (*W_d_*) of the dried gels was measured. Water content was determined using Equation (1):

Water content (%) = (*W_s_* − *W_d_*)/*W_s_* × 100 (1)

### 2.6. Observation of Polymer Network Structure in Gel Using Scanning Electron Microscope

The cross-sectional morphology of each gel was observed using a scanning electron microscope (JCM-6000, JEOL, Tokyo, Japan). GC-ACG and DN gels were prepared and freeze-dried at −80 °C to volatilize water. The samples were then cut and sputtered with a thin gold layer. Their morphologies were detected at 15 kV.

### 2.7. Reductive Amination for GC-ACG

In order to restrain reversible formation and dissociation of Schiff base, GC-ACG was soaked in a 1.5 M aqueous solution of NaBH_3_CN at 30 °C for 24 h for reductive amination (RA) to convert the Schiff base into the corresponding amine in the gel. After the RA treatment, the gel was immersed in water for 1 d to remove unreacted NaBH_3_CN and its by-products. Then, the PDMAAm network was formed in this gel using the method described in Section 2.3.

### 2.8. Examination of Cell Adhesion on Gels

The V79 cell line was cultured in MEM containing 10% inactivated FBS, 2 mM L-glutamine, and ×1 Penicilin/Streptomycin. NHDF cells were cultured in the FibroLife comp kit. The V79 cell line is used within five passages, and NHDF cell is used within three passages.

DN gels before and after reductive amination (RA) treatment were cut out in a round column using a biopsy punch (φ8 mm KAI Medical, Gifu, Japan). Each was placed individually on a well of a 48-well plate. Cells (2.5 × 10^4^) of V79 cell line or NHDF were seeded on each gel and cultured at 37 °C in an incubator (humidified 95% air, 5% CO_2_). After 24 h, the culture medium was replaced with another medium containing 1 μM calcein-AM, and the cells were incubated for 20 min. Fluorescent images of living cells were obtained using imaging software (cellSens Standard ver1.13, OLYMPUS Co., Tokyo, Japan) and a fluorescence microscope (IX71, OLYMPUS Co.).

### 2.9. Statistical Analysis

Data are expressed as mean ± standard deviation. Statistical comparisons were performed using Student’s *t*-test. Differences were statistically significant at *p* < 0.05. All error bars represent standard deviations.

## 3. Results and Discussion

### 3.1. Mechanical Properties of GC-ACG

First, we prepared GC-ACG and evaluated its mechanical properties using a tensile test. Figure 2a shows a representative photograph of the tensile test setup. The stress–strain curve of GC-ACG prepared with various GA concentrations is shown in Figure 2b. The major mechanical values were calculated from this stress–strain curve (Figure 2c–e). Young’s modulus was determined from the initial slope of this stress–strain curve. These results indicate that both the fracture stress and Young’s modulus increased, and the fracture strain decreased as the GA concentration in the pre-gel solution increased. The observed changes in the mechanical properties are consistent with those arising from typical chemically cross-linked hydrogels. Although stiffer gels can be prepared by increasing the cross-linking density, the distances between the cross-linked points in the polymer network become shorter, and the mechanical properties become brittle. Therefore, stiff and brittle gels were prepared by varying the cross-linker content in the pre-gel solution; however, a tough hydrogel, which improved both the fracture stress and strain, could not be developed through simple changes in the cross-linker content. Upon increasing the concentration of GA, the fracture stress increased, and the fracture strain decreased; however, the values did not increase or decrease significantly at molar ratios (GA/(Lys and Hyl)) higher than 30 (Figure 2c,d). This result indicates that all reactive residues in atelocollagen molecules reacted with the aldehyde group in GA at GA/(Lys and Hyl) = 30, and no further change in the mechanical properties was observed even when the GA content was higher. As mentioned in the Introduction, we selected GC-ACG as a stiff and brittle polymer network to develop a tough composite gel based on the DN gel principle. Thus, for the pre-gel solution, we chose GA/(Lys and Hyl) = 30, which is 0.2 M GA, and prepared DN gels in the subsequent section.

### 3.2. Comparison of Characteristics between GC-ACG and Composite Gel

**Figure 3:**
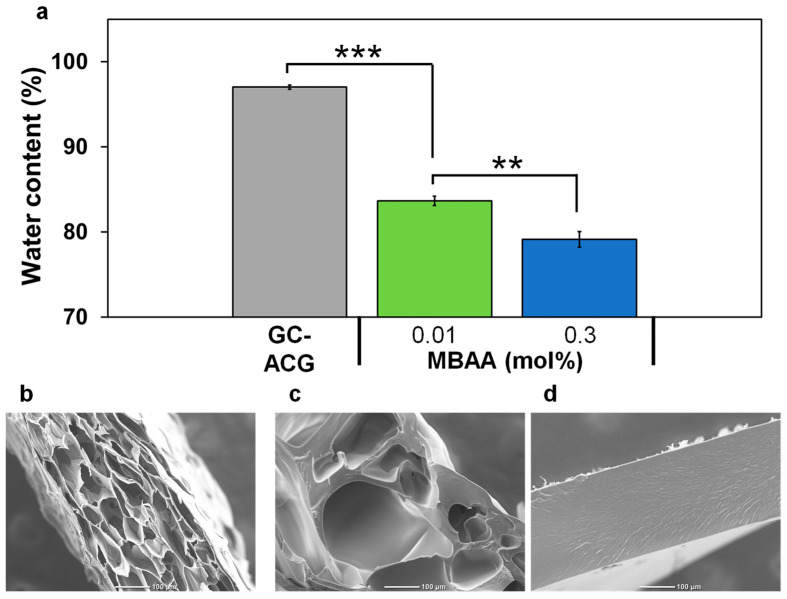
Characteristics of composite gels. (**a**) Water content in atelocollagen gel (GC-ACG) and composite gels prepared by two *N*,*N’*-methylenebisacrylamide (MBAA) concentrations (0.01 and 0.3 mol%, n = 3, ** *p* < 0.01, *** *p* < 0.001). (**b**–**d**) Scanning electron microscopy (SEM) images of GC-ACG (**b**) and DN gels prepared by two MBAA concentrations (0.01 mol% (**c**) and 0.3 mol% (**d**)).

According to the scheme illustrated in Figure 1, we prepared composite gels by combining the GC-ACG and cross-linked PDMAAm gels. First, we measured the water content of both the GC-ACG and composite gels to clarify the water swelling capacity of the gel. We prepared GC-ACG using a pre-gel solution containing 2 wt% atelocollagen and 0.2 M GA, as mentioned above, and subsequently formed the PDMAAm network in this gel. As shown in Figure 3a, the water content of the composite gel was lower than that of the GC-ACG. Previous research on DN gels also revealed that the water content was diminished by combining with another polymer network [22]. Atelocollagen and PDMAAm are highly hydrophilic polymers; therefore, the composite gel can swell in water. Considering the interaction in the composite gel between the hydrophilic group of atelocollagen molecules and PDMAAm, dipole interactions such as hydrogen bonds and van der Waals attraction can occur because the two polymer networks are interpenetrated and their distance becomes closer compared to a single network. Therefore, we speculated that the number of water molecules interacting with atelocollagen or PDMAAm decreased, lowering the water content retained in the composite gel, even though these polymers are hydrophilic. Moreover, the water content of the composite gel prepared using 0.3 mol% MBAA was lower than that prepared using 0.01 mol% MBAA. Generally, it is well known that the water content decreases as the cross-linking density increases based on the rubber elasticity of the gel. Thus, the water content of the composite gel decreases as the MBAA concentration increases. The change in water content of the gel by combining PDMAAm is consistent with the characteristics of the DN gel. Moreover, we observed cross-sectional morphology using a scanning electron microscope to visualize the internal microstructure of the prepared composite gel. Figure 3b–d show scanning electron microscopy (SEM) images of GC-ACG and DN gels prepared using 0.01 or 0.3 mol% MBAA. Several large pores were observed in the cross-section of GC-ACG, and the skeleton of the atelocollagen network was very thin with a porous structure. In contrast, in the composite gel prepared using 0.01 mol% MBAA, the skeleton was thicker. This result is similar to the SEM images reported in previous DN gel studies [34]. Moreover, in the composite gel prepared using higher MBAA, 0.3 mol%, such pores were not observed, and a smooth surface appeared, indicating that a dense structure was formed in the gel. This structure seems to be aggregated not only in atelocollagen but also in the PDMAAm network. Considering the results of the water content depicted in the former together, these SEM images indicate that the two polymer networks combined as expected, and the objective composite gel is considered to be strongly identical to the DN gel, as reported in previous research. In the subsequent section, we use the word “DN gel” as the objective composite gel.

### 3.3. Mechanical Properties of DN Gels Composed of Atelocollagen and PDMAAm

**Figure 4:**
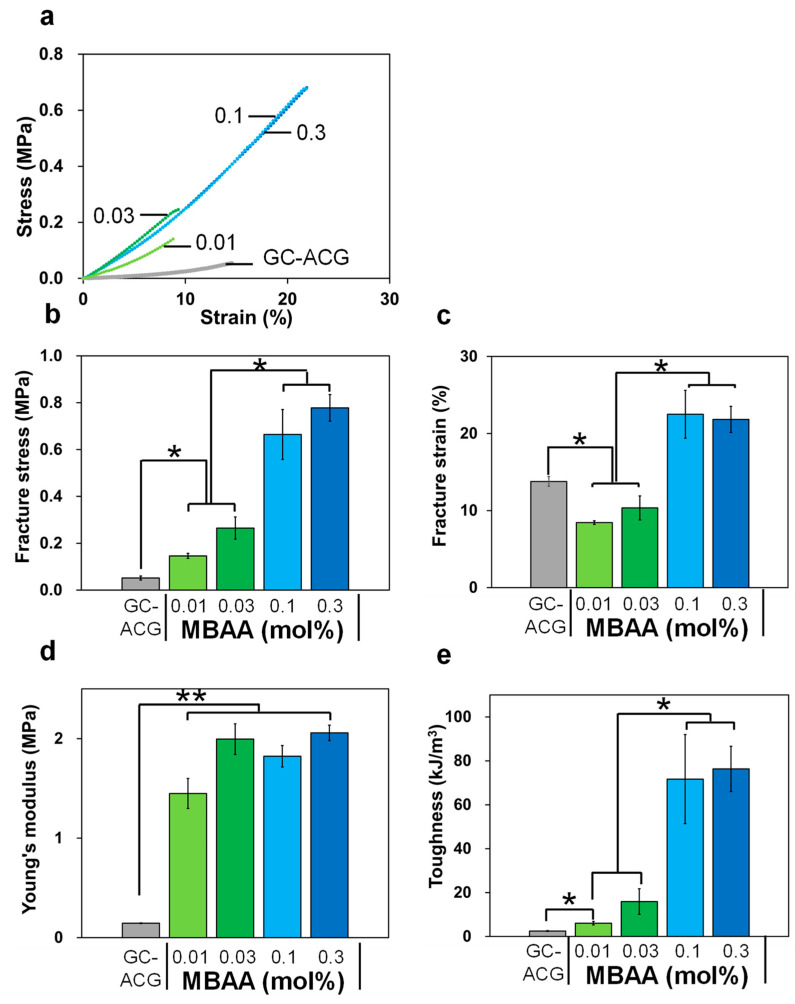
Mechanical tensile properties of DN atelocollagen gels: (**a**) stress–strain curve; (**b**) fracture stress; (**c**) fracture strain; (**d**) Young’ modulus; (**e**) toughness (n = 3; * *p* < 0.05; ** *p* < 0.01).

We evaluated their mechanical properties using the method described in Section 3.1 to confirm the enhanced toughness of the prepared DN gels. The representative stress–strain curves of DN gels composed of PDMAAm and atelocollagen prepared at various MBAA concentrations are shown in Figure 4a. From this result, each mechanical value is calculated (Figure 4b–e). Toughness is the energy required to fracture a material and is normalized by its volume. Compared with GC-ACG, the fracture stress, Young’s modulus, and toughness of the DN gels were much higher for all MBAA concentrations. This enhancement of mechanical properties is attributed to effective energy dissipation through the combination of two polymer networks, which has been reported in previous research on DN gels [22,23,25]. The fracture strain on less than 0.03 mol% MBAA was lower than that on GC-ACG, whereas it was higher on more than 0.1 mol% MBAA. Moreover, between less than 0.03 mol% and more than 0.1 mol% MBAA, a significant difference was confirmed in fracture stress, fracture strain, and toughness. The result suggests that enough cross-linking density of the PDMAAm network is essential to enhance the toughness of DN gels. As shown in Figure 3, water content in the DN gel at MBAA 0.3 mol% was lower than that at MBAA 0.01 mol%; in other words, the polymer density was higher at MBAA 0.3 mol%. This difference in polymer density may affect the internal microstructure observed by SEM and consequently enhance toughness. As shown in Figure 4b, the fracture stress at MBAA 0.3 mol% was 0.8 MPa. Other recent reports on tough gels showed that their tensile fracture stress was several hundred kPa [30,31] or more than 1 MPa [18], which is comparable to that of our developed DN gels. Furthermore, the latest studies on tough gels focus on physical polymer entanglement in gels, aiming to enhance the viscous characteristics for energy dissipation [30,31,32]. Norioka et al. showed that tough poly-(acrylamide) hydrogels can be prepared by free radical polymerization with a high monomer concentration and low cross-linker content to induce polymer chain entanglements for energy dissipation. This method can be applied to our developed DN gels for toughness enhancement because the PDMAAm network forming the DN gels was also prepared by free radical polymerization. In the future, we will clarify the mechanical properties of DN gels prepared with higher DMAAm concentrations and lower MBAA concentrations.

### 3.4. Cell Adhesion on DN Gels Treated via RA

**Figure 5:**
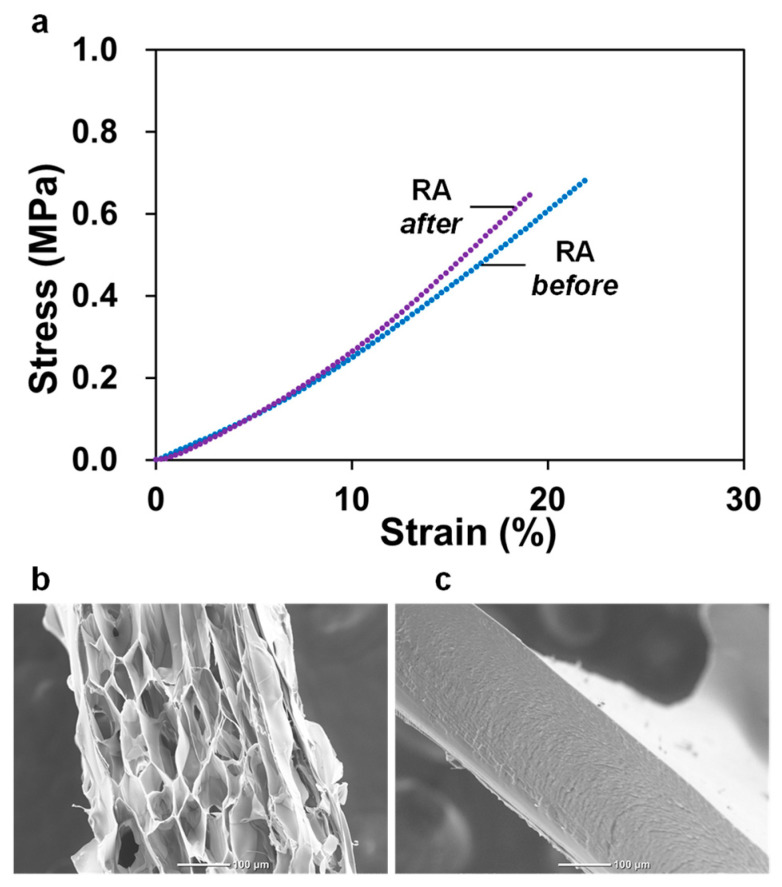
Characteristics of the developed double-network (DN) gel after reductive amination (RA) treatment. (**a**) Tensile stress–strain curve of DN gels prepared using 0.3 mol% *N*,*N’*-methylenebisacrylamide (MBAA) before or after RA treatment. (**b**) Scanning electron microscopy (SEM) image of atelocollagen gel. (**c**) SEM image of the DN gel prepared using 0.3 mol% MBAA after RA treatment.

Finally, we evaluated the cell adhesive properties of DN gels to consider their in vivo and in vitro research of the objective. GA is known as a cross-linking agent with high cytotoxicity, although it has been the most widely used in collagen gel research. It is because the Schiff base formed between GA and the amino group in collagen can be reversibly hydrolyzed in the presence of water, and the free GA exhibits cytotoxic effects. Therefore, a method for preventing the hydrolysis of the Schiff base is essential for its bio-application. We then selected the RA approach via lysine residues of atelocollagen and the aldehyde groups of GA using the method described in Section 2.7. This treatment can prohibit cytotoxic GA from diffusing into the culture medium, which allows cells to adhere and grow on the gel. This process is very simple, and the reaction can be generated in water. The RA-treated GC-ACG needs to be washed sufficiently to remove unreacted NaBH_3_CN and its by-products existing in the gel because the NaBH_3_CN itself is a toxic substance. However, NaBH_3_CN, which is a water-soluble substance, can be removed easily with water. We prepared GC-ACGs treated with RA and DN gels by combining the PDMAAm network. We further confirmed that the cells adhered on the DN gel-treated RA. As shown in Figure 5, RA did not cause significant changes in the mechanical properties and microstructures of the DN gels.

**Figure 6:**
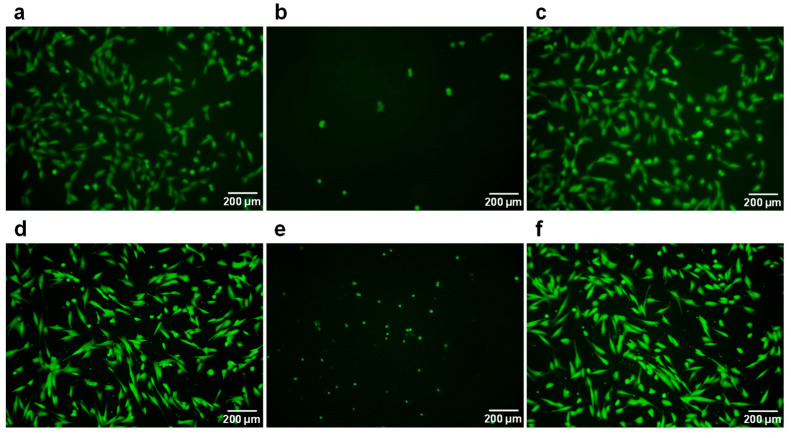
Images of cell adhesion on cell culture treated polystyrene (PS) substrate and DN gels ((**a**–**c**): V79 cell line; (**d**–**f**): Primary Normal Human Dermal Fibroblast (NHDF) cell). (**a**,**d**) cell culture-treated PS substrates; (**b**,**e**) the DN gel before reductive amination (RA) treatment; (**c**,**f**) the DN gel after RA treatment.

Figure 6 shows the images of the V79 cell line and NHDF cells on cell culture-treated PS substrate and DN gels before and after RA treatment. We used the V79 cell line and NHDF cells in this study because the V79 cell line has been used to evaluate the cytotoxicity of medical equipment [43,44,45], and NHDF cells are dermal fibroblasts derived from humans. As shown in Figure 6, the V79 cell line is suspended above the gel before RA treatment without sufficient cell adhesion. Conversely, they adhered and spread on the gel after RA treatment, likewise on the PS substrate. Moreover, no significant difference in cell morphology was observed between cell culture-treated PS substrate and DN gel after RA treatment. A similar result was obtained when NHDF cells were seeded. These results indicate that RA treatment can relieve the cytotoxicity of GA. Consequently, regardless of the cell species, RA treatment enables cells to adhere and spread on the toughness-enhanced DN gel almost as much as on typical PS-based cell culture substrates. RA treatment overcame the problem of GA’s cytotoxicity, which has been a concern for a long time, and the result is worthwhile for biomedical applications using collagen materials. The developed DN gel, composed of atelocollagen and a biocompatible polymer, has the potential to be applied as an artificial alternative material to soft tissues in the human body. Especially because the DN gel showed tensile fracture stress of sub-MPa, as represented in Figure 4, it can be applied to soft tissues whose fracture stress value is similar, such as the aorta [46]. However, representative soft tissues having high demand and difficulty for their alternative materials are tendons and ligaments owing to their high tensile strength. Indeed, a previous report shows that fracture stress of the human anterior cruciate ligament was approximately 24 MPa [47]. In order to enhance the mechanical properties of the DN gel, the construction of uniform orientation of collagen fibrils using a microfluidic system [48] is a useful technique for imitation of actual tendon or ligament internal structure. Aiming for the realization of alternative materials for such tough tissues, further investigation of the mechanical and biochemical properties of the DN gel needs to be conducted in the future to improve the feasibility of the DN gel.

## 4. Conclusions

In this study, we developed composite gels composed of atelocollagen with reduced immunogenicity and PDMAAm, a biocompatible polymer based on the DN gel principle. Compared to GC-ACG, the water content decreased, and the internal structure became denser, suggesting that DN gel was formed in the same way as previous research. Tensile tests revealed that the toughness of the DN gel was approximately 30 times higher than that of GC-ACG, and the fracture stress was comparable to that of some recently reported tough gels. Moreover, both V79 and NHDF cells adhered and spread on the DN gel after RA treatment, even though they did not adhere on the gel before RA treatment. Above all, this tough gel material using atelocollagen is thought to be a promising biomaterial, such as an artificial alternative material to soft tissue in the human body.

## Data Availability

Data are contained within the article.

## References

[1] Takezawa T. (2021). Revolution in Cell Culture Technology Based on a Novel Concept for Enclosing Cells using Collagen Vitrigel Membranes. Nano Biomed..

[2] Oshikata-Miyazaki A., Takezawa T. (2016). Development of an oxygenation culture method for activating the liver-specific functions of HepG2 cells utilizing a collagen vitrigel membrane chamber. Cytotechnology.

[3] Friess W. (1998). Collagen—Biomaterial for drug delivery. Eur. J. Pharm. Biophram..

[4] Lynn A.K., Yannas I.V., Bonfield W. (2004). Antigenicity and immunogenicity of collagen. J. Biomed. Mater. Res. B Appl. Biomater..

[5] Sano A., Maeda M., Nagahara S., Ochiya T., Honma K., Itoh H., Miyata T., Fujioka K. (2003). Atelocollagen for protein and gene delivery. Adv. Drug Deliv. Rev..

[6] Ogawa S., Onodera J., Honda R., Fujimoto I. (2011). Influence of systemic administration of atelocollagen on mouse livers: An ideal biomaterial for systemic drug delivery. J. Toxicol. Sci..

[7] Fujimoto I., Takei Y. (2014). Atelocollagen-mediated SiRNA delivery: Future promise for therapeutic application. Ther. Deliv..

[8] Suzuki R., Nakamura R., Nakaegawa Y., Nomoto Y., Fujimoto I., Semura K., Hazama A., Omori K. (2016). Optimal bovine collagen concentration to achieve tracheal epithelial coverage of collagen sponges. Laryngoscope.

[9] Sato T., Semura K., Fujimoto I. (2019). Micro-dimpled surface atelocollagen maintains primary human hepatocytes in culture and may promote their functionality compared with collagen coat culture. Int. J. Mol. Med..

[10] Kubota R., Fujimoto I. (2023). Synthesis, Characterization, and Potential Application of Cyclodextrin-Based Polyrotaxanes for Reinforced Atelocollagen Threads. Polymers.

[11] Kubota R., Naritomi M., Fujimoto I. (2023). Synthesis of a stretchable polymer crosslinker for reinforced atelocollagen threads. React. Funct. Polym..

[12] Hoffman A.S. (2012). Hydrogels for biomedical applications. Adv. Drug Deliv. Rev..

[13] Peppas N.A., Hilt J.Z., Khademhosseini A., Langer R. (2006). Hydrogels in Biology and Medicine: From Molecular Principles to Bionanotechnology. Adv. Mater..

[14] Li J., Mooney D.J. (2016). Designing hydrogels for controlled drug delivery. Nat. Rev. Mater..

[15] Zhang Y.S., Khademhosseini A. (2017). Advances in engineering hydrogels. Science.

[16] Okumura Y., Ito K. (2001). The Polyrotaxane Gel: A Topological Gel by Figure-of-Eight Cross-links. Adv. Mater..

[17] Noda Y., Hayashi Y., Ito K. (2014). From Topological Gels to Slide-Ring Materials. J. Appl. Polym. Sci..

[18] Liu C., Morimoto N., Jiang L., Kawahara S., Noritomi T., Yokoyama H., Mayumi K., Ito K. (2021). Tough hydrogels with rapid self-reinforcement. Science.

[19] Haraguchi K., Takehisa T. (2002). Nanocomposite Hydrogels: A Unique Organic-Inorganic Network Structure with Extraordinary Mechanical, Optical, and Swelling/De-swelling Properties. Adv. Mater..

[20] Haraguchi K. (2007). Nanocomposite hydrogels. Curr. Opin. Solid State Mater. Sci..

[21] Sakai T., Matsunaga T., Yamamoto Y., Ito C., Yoshida R., Suzuki S., Sasaki N., Shibayama M., Chung U.-I. (2008). Design and Fabrication of a High-Strength Hydrogel with Ideally Homogeneous Network Structure from Tetrahedron-like Macromonomers. Macromolecules.

[22] Gong J.P., Katsuyama Y., Kurokawa T., Osada Y. (2003). Double-Network Hydrogels with Extremely High Mechanical Strength. Adv. Mater..

[23] Gong J.P. (2010). Why Are Double Network Hydrogels so Tough?. Soft Matter.

[24] Nakajima T., Sato H., Zhao Y., Kawahara S., Kurokawa T., Sugahara K., Gong J.P. (2012). A Universal Molecular Stent Method to Toughen Any Hydrogels Based on Double Network Concept. Adv. Funct. Mater..

[25] Nakajima T. (2017). Generalization of the sacrificial bond principle for gel and elastomer toughening. Polym. J..

[26] Mredha M.T.I., Kitamura N., Nonoyama T., Wada S., Goto K., Zhang X., Nakajima T., Kurokawa T., Takagi Y., Yasuda K. (2017). Anisotropic tough double network hydrogel from fish collagen and its spontaneous in vivo bonding to bone. Biomaterials.

[27] Sun J.-Y., Zhao X., Illeperuma W.R.K., Chaudhuri O., Oh K.H., Mooney D.J., Vlassak J.J., Suo Z. (2012). Highly stretchable and tough hydrogels. Nature.

[28] Sun T.L., Kurokawa T., Kuroda S., Ihsan A.B., Akasaki T., Sato K., Haque M.A., Nakajima T., Gong J.P. (2013). Physical hydrogels composed of polyampholytes demonstrate high toughness and viscoelasticity. Nat. Mater..

[29] Matsuda T., Kawakami R., Namba R., Nakajima T., Gong J.P. (2019). Mechanoresponsive self-growing hydrogels inspired by muscle training. Science.

[30] Norioka C., Inamoto Y., Hajime C., Kawamura A., Miyata T. (2021). A universal method to easily design tough and stretchable hydrogels. NPG Asia Mater..

[31] Kim J., Zhang G., Shi M., Suo Z. (2021). Fracture, fatigue, and friction of polymers in which entanglements greatly outnumber cross-links. Science.

[32] Nian G., Kim J., Bao X., Suo Z. (2022). Making Highly Elastic and Tough Hydrogels from Doughs. Adv. Mater..

[33] Fujiyabu T., Sakumichi N., Katashima T., Liu C., Mayumi K., Chung U.-I., Sakai T. (2022). Tri-branched gels: Rubbery materials with the lowest branching factor approach the ideal elastic limit. Sci. Adv..

[34] Chen J.X., Yuan J., Wu Y.L., Wang P., Zhao P., Lv G.Z., Chen J.H. (2018). Fabrication of tough poly(ethylene glycol)/collagen double network hydrogels for tissue engineering. J. Biomed. Mater. Res. A.

[35] Hanyková L., Krakovský I., Šestáková E., Šťastná J., Labuta J. (2020). Poly(*N*,*N′*-Diethylacrylamide)-Based Thermoresponsive Hydrogels with Double Network Structure. Polymers.

[36] Lee H., Hong H.J., Ahn S., Kim D., Kang S.H., Cho K., Koh W.G. (2023). One-Pot Synthesis of Double-Network PEG/Collagen Hydrogel for Enhanced Adipogenic Differentiation and Retrieval of Adipose-Derived Stem Cells. Polymers.

[37] Olăreț E., Bălănucă B., Onaș A.M., Ghițman J., Iovu H., Stancu I.C., Serafim A. (2021). Double-Cross-Linked Networks Based on Methacryloyl Mucin. Polymers.

[38] Cong J., Fan Z., Pan S., Tian J., Lian W., Li S., Wang S., Zheng D., Miao C., Ding W. (2021). Polyacrylamide/Chitosan-Based Conductive Double Network Hydrogels with Outstanding Electrical and Mechanical Performance at Low Temperatures. ACS Appl. Mater. Interfaces.

[39] Zhang H., Shi L.W.E., Zhou J. (2023). Recent Developments of Polysaccharide-Based Double-Network Hydrogels. J. Polym. Sci..

[40] Zhang W., Chen S., Jiang W., Zhang Q., Liu N., Wang Z., Li Z., Zhang D. (2023). Double-Network Hydrogels for Biomaterials: Structure-Property Relationships and Drug Delivery. Eur. Polym. J..

[41] Sung H.-W., Huang R.-N., Huang L.L.H., Tsai C.-C., Chiu C.-T. (1998). Feasibility Study of a Natural Crosslinking Reagent for Biological Tissue Fixation. J. Biomed. Mater. Res..

[42] Damink L.H.H.O., Dijkstra P.J., Van Luyn M.J.A., Van Wachem P.B., Nieuwenhuis P., Feijen J. (1995). Glutaraldehyde as a crosslinking agent for collagen-based biomaterials. J. Mater. Sci. Mater. Med..

[43] Yonezawa K., Kawaguchi M., Kaneuji A., Ichiseki T., Iinuma Y., Kawamura K., Shintani K., Oda S., Taki M., Kawahara N. (2020). Evaluation of Antibacterial and Cytotoxic Properties of a Fluorinated Diamond-like Carbon Coating for the Development of Antibacterial Medical Implants. Antibiotics.

[44] Saito N., Haniu H., Usui Y., Aoki K., Hara K., Takanashi S., Shimizu M., Narita N., Okamoto M., Kobayashi S. (2014). Safe Clinical Use of Carbon Nanotubes as Innovative Biomaterials. Chem. Rev..

[45] Kavasi R.-M., Coelho C.C., Platania V., Quadros P.A., Chatzinikolaidou M. (2021). In Vitro Biocompatibility Assessment of Nano-hydroxyapatite. Nanomaterials.

[46] Haut R.C., Nahum A.M., Melvin J.W. (2002). Biomechanics of Soft Tissue. Accidental Injury: Biomechanics and Prevention.

[47] Chandrashekar N., Mansouri H., Slauterbeck J., Hashemi J. (2006). Sex-Based Differences in the Tensile Properties of the Human Anterior Cruciate Ligament. J. Biomech..

[48] Haynl C., Hofmann E., Pawar K., Förster S., Scheibel T. (2016). Microfluidics-Produced Collagen Fibers Show Extraordinary Mechanical Properties. Nano Lett..

